# Computational wave-based photoacoustic imaging through an unknown thick aberrating layer

**DOI:** 10.1016/j.pacs.2024.100584

**Published:** 2024-01-19

**Authors:** Yevgeny Slobodkin, Ori Katz

**Affiliations:** Institute of Applied Physics, The Hebrew University of Jerusalem, Jerusalem 9190401, Israel

**Keywords:** Photoacoustic tomography, Aberration correction, Model-based reconstruction, Beamforming, Iterative optimization

## Abstract

We introduce a physics-based computational reconstruction framework for non-invasive photoacoustic tomography through a thick aberrating layer. Our wave-based approach leverages an analytic formulation of diffraction to beamform a photoacoustic image, when the aberrating layer profile is known. When the profile of the aberrating layer is unknown, the same analytical formulation serves as the basis for an automatic-differentiation regularized optimization algorithm that simultaneously reconstructs both the profile of the aberrating layer and the optically absorbing targets. Results from numerical studies and proof-of-concept experiments show promise for fast beamforming that takes into account diffraction effects occurring in the propagation through thick, highly-aberrating layers.

## 1 Introduction

Photoacoustic (PA) imaging is the state-of-the-art deep tissue imaging technique that combines light and sound, but may be limited in resolution when acoustic aberrations are present. The reason that aberrations affect PA reconstructed images is that speed-of-sound may vary relatively largely in biological samples, leading to distortions in the recorded ultrasound signals, thus degrading the effective imaging resolution. In the past two decades, there have been many efforts in undoing the effect of acoustic aberrations [1], [2], [3], [4], [5], [6], [7], [8], [9], [10], [11], [12], [13], [14], [15]. When the variations in the speed-of-sound are small (below 10%), as is largely the case in soft tissues, refraction is negligible, and several modified reconstruction techniques, based on compensating for the delays in time-of-flight of the recorded ultrasound waves while neglecting refraction, can be effectively applied [12], [13], [14], [15]. For scenarios involving significant variations in the speed-of-sound, full-wave model-based algorithms can, in principle, accommodate any effect associated with acoustic propagation in strongly mismatched tissues. However, this strategy demands precise prior knowledge of the distribution of acoustic properties, and very computationally intensive calculations [16]. Alternatively, if the photoacoustic targets are localized within the usually small isoplanatic patch (or memory effect) and a reference signal from a point-like source (a guide star) is available, memory-effect-based reconstruction becomes feasible [16], [17].

**Fig. 1:**
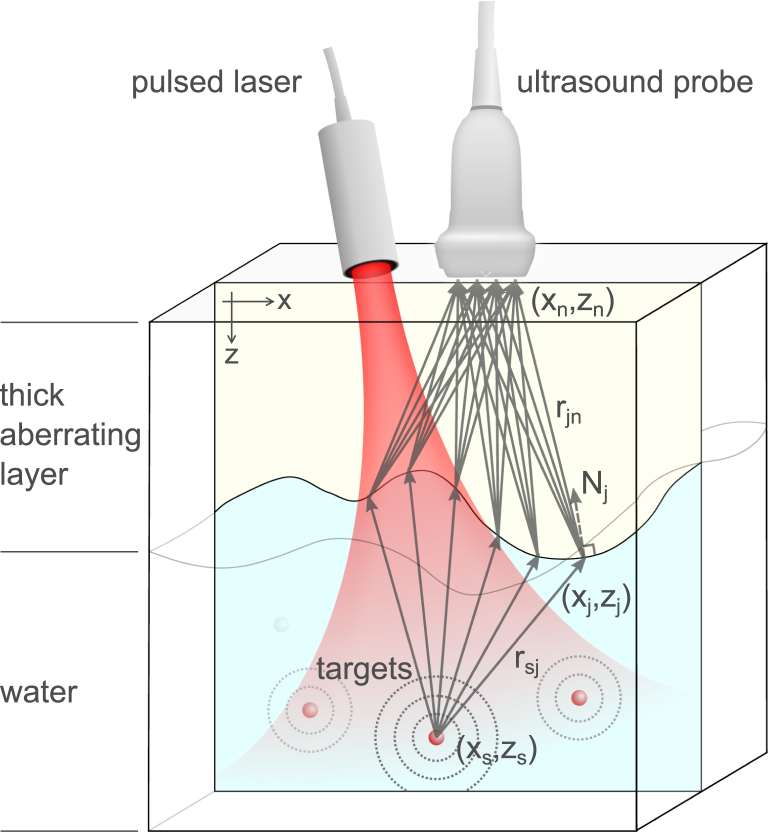
Photoacoustic imaging through a thick aberrating layer — illustration of the problem. A laser pulse (red beam) illuminates optically absorbing targets (red spheres), leading to local increase in pressure and temperature. Acoustic waves (dashed circles) propagate from the absorbing targets to an ultrasound probe. The difference in speed of sound between the aberrating layer and water results in time delays and distortions of the measured ultrasound waves. For each location of an absorber in the media, acoustic waves, originating at $\left(x_{s},z_{s}\right)$, undergo a single refraction at every point along the interface between the aberrator and water $\left(x_{j},z_{j}\right)$. Further propagation through the aberrator induces time delays to the acoustic waves, before they interfere at each point at the sensor $\left(x_{n},z_{n}\right)$. The presented physical model beam-former is based on the Fresnel–Kirchhoff diffraction formula for wave propagation [18], [19], [20], where every point along the aberrator-water medium is itself the source of spherical wavelets (illustrated by solid arrows). An unknown aberrator profile can be reconstructed together with the absorbers locations through iterative regularized optimization.

In cases such as transcranial imaging of the brain, fetal ultrasound and ultrasound imaging of obese patients, a thick acoustically aberrating layer is located near the transducer array (Fig. 1), leading to distortions in the recorded signal. In such cases, a delay-and-sum (DAS) algorithm with the assumption of a spatially constant speed-of-sound would lead to incorrect images of the target. Previous works, which modeled heterogeneous media as layered structures, have employed ray-based models where refraction is taken into account by applying Snell’s law [21], [22]. These models require the precise prior knowledge of the thickness profile of the aberrating layers, and operate under the assumption that the wavelength is considerably smaller than all other spatial dimensions of the problem. In situations such as wave propagation in the skull, the acoustic wavelength can be comparable to or even larger than the thickness of the skull, leading to a scenario where diffraction, rather than ray theory, is dominant [21], [23].

In this work, we present a novel computational framework specifically designed for photoacoustic imaging through a thick and highly-aberrating layer, based on Fresnel–Kirchhoff diffraction model for wave propagation that accounts for temporal and spatial distortions. Our approach takes into account refraction and diffraction, at a significantly lower computational cost than full-wave approaches.

When the exact geometry of the aberrating layer is known, reconstruction is performed by Fresnel–Kirchhoff based modified beamforming, taking into account diffraction, refraction and interference, which is able to compensate for the results of aberrations. This beamforming approach leads to a more reliable reconstruction compared to conventional beamforming models that neglect refraction [12], [13], [14], [15], while being computationally efficient compared to a full-wave iterative reconstruction with acoustically inhomogeneous media [11], [24].

When the shape of the aberrating layer is unknown, we demonstrate that the exact geometry of the aberrating layer can be found through a model-based iterative optimization. We demonstrate that, through automatic differentiation of the diffraction-based model, a gradient descent algorithm can simultaneously find the target and aberrator estimates that: (1) best fit the measured signals, and (2) obey a chosen prior information on the target and aberrator, used as a regularizer for the reconstruction.

## 2 Material and methods

### 2.1 Forward model

Our physical model for wave propagation in inhomogeneous media is based on the Fresnel–Kirchhoff diffraction integral [18], [19], [20], which provides an integral expression for the diffraction of a spherical wave passing through an opening in an opaque screen. This diffraction formula manifests the Huygens principle, where every point along the wavefront over the opening can be considered as a source of a spherical wavelet. The interference of the multiple wavelets produces the transmitted diffraction pattern. Using this approach to calculate the propagation of a wave through an aberrating ‘opening’ is advantageous as it naturally takes into account in a computationally efficient manner interference effects such as refraction and diffraction, which are conventionally neglected when using a standard DAS reconstruction.

In our model we assume that the medium can be divided into two regions with two different (but constant) sound velocities- $c_{w}$ for water and $c_{a}$ for the aberrating layer, as illustrated in Fig. 1. We assume that the target is surrounded by a homogeneous medium (water), and that the acoustic waves undergo a single refraction and phase delays by passing through the aberrating layer interface before reaching the sensor. Under these assumptions, the pressure $p\left({\vec{r}}_{n},t\right)$ recorded at the sensor element n, positioned at ${\vec{r}}_{n}$, from a set of point absorbers located at ${\vec{r}}_{s}$ and photoacoustically excited at time t=0 can be obtained by modifying the Fresnel–Kirchhoff diffraction formula to yield [18], [19], [20] Appendix A:

(1) $$p\left({\vec{r}}_{n},t\right)\propto \underset{s}{\sum }\underset{j}{\sum }\frac{a_{s}\psi_{sjn}}{r_{sj}r_{jn}}h_{n}\left(t-\frac{r_{sj}}{c_{w}}-\frac{r_{jn}}{c_{a}}\right)$$

Here $\sum_{s}$ represents the sum over all acoustic point absorbers. For each point absorber, $\sum_{j}$ is the sum of the contributions of the Huygens wavelets from all points over the aberrator surface (Fig. 1). $a_{s}$ is the initial pressure amplitude at point ${\vec{r}}_{s}$. $r_{sj}$ is the distance from point ${\vec{r}}_{s}$ (the acoustic source) to point ${\vec{r}}_{j}$ (the aberrator surface). Similarly, $r_{jn}$ is the distance from point ${\vec{r}}_{j}$ (the aberrator surface) to point ${\vec{r}}_{n}$ (the transducer element). $h_{n}\left(t\right)$ is the effective overall temporal impulse-response of sensor element n to pulsed optical excitation of a point absorber. $\psi_{sjn}$ is known as the obliquity factor [18]. According to the Fresnel–Kirchhoff theory, the obliquity factor $\psi_{sjn}$ in homogeneous media introduces an angular dependence via two cosine terms, and can be expressed as $\psi_{sjn}^{\mathrm{Kirchhoff}}\propto {\overset{ˆ}{N}}_{j}\cdot {\overset{ˆ}{r}}_{sj}+{\overset{ˆ}{N}}_{j}\cdot {\overset{ˆ}{r}}_{jn}$, where ${\overset{ˆ}{N}}_{j}$ is the normal to the aberrator’s surface at point j pointing inwards towards the probe (Fig. 1), such that the term ${\overset{ˆ}{N}}_{j}\cdot {\overset{ˆ}{r}}_{sj}=\cos\phi_{in}$ is cosine the angle of incidence, and ${\overset{ˆ}{N}}_{j}\cdot {\overset{ˆ}{r}}_{jn}=\cos\phi_{out}$ is the cosine angle between the direction of propagation inside the aberrator and the normal to the aberrator’s surface. Adapting Kirchhoff theory specifically to the case of a thick aberrating layer, we obtain $\psi_{sjn}\propto {\overset{ˆ}{N}}_{j}\cdot {\overset{ˆ}{r}}_{sj}/c_{w}+{\overset{ˆ}{N}}_{j}\cdot {\overset{ˆ}{r}}_{jn}/c_{a}$ (Appendix A). Alternative formulation of the forward model can be obtained based on the Rayleigh-Sommerfeld diffraction formula [18], leading to $\psi_{sjn}^{\mathrm{Sommerfeld,I}}={\overset{ˆ}{N}}_{j}\cdot {\overset{ˆ}{r}}_{jn}=\cos\phi_{out}$ and $\psi_{sjn}^{\mathrm{Sommerfeld,II}}={\overset{ˆ}{N}}_{j}\cdot {\overset{ˆ}{r}}_{sj}=\cos\phi_{in}$ for the first and second Rayleigh-Sommerfeld solutions, respectively. We note that, while the different obliquity factors represent different angular dependencies, nearly identical numerical and experimental results were obtained with each of the above formulations of ψ, as well as for setting ψ=1. Additional physical effects, such as absorption and wave conversion, are not considered in the proposed forward model, in order to emphasize the detrimental effects of refraction and diffraction alone in thick aberrating layers. Importantly, such physical effects can be seamlessly incorporated into the forward model. For example, frequency-dependent absorption coefficients in water, $\alpha_{w}\left(f\right)$, and the aberrating layer, $\alpha_{a}\left(f\right)$, can both be added to the forward model by multiplying each wavelet that originates from the source ${\vec{r}}_{s}$, refracts at ${\vec{r}}_{j}$ and reaches the sensor at ${\vec{r}}_{n}$ by the corresponding absorption factor, $\exp\left[-\alpha_{w}\left(f\right)r_{sj}-\alpha_{a}\left(f\right)r_{jn}\right]$.

### 2.2 Beamforming models

When the exact geometry of the aberrating layer is known or measured, back-projection of Eq. (1) can be used to reconstruct the initial pressure distribution $A\left({\vec{r}}_{s}\right)$:

(2) $$A\left({\vec{r}}_{s}\right)\propto \underset{n}{\sum }\underset{j}{\sum }\frac{r_{sj}r_{jn}}{\psi_{sjn}}p\left({\vec{r}}_{n},t=\frac{r_{sj}}{c_{w}}+\frac{r_{jn}}{c_{a}}\right)$$

In comparison, conventional DAS beamforming can be expressed as:

(3) $$A^{\mathrm{conv.}}\left({\vec{r}}_{s}\right)\propto \underset{n}{\sum }r_{sn}p\left({\vec{r}}_{n},t=\frac{r_{sn}}{c_{w}}\right)$$

**Fig. 2:**
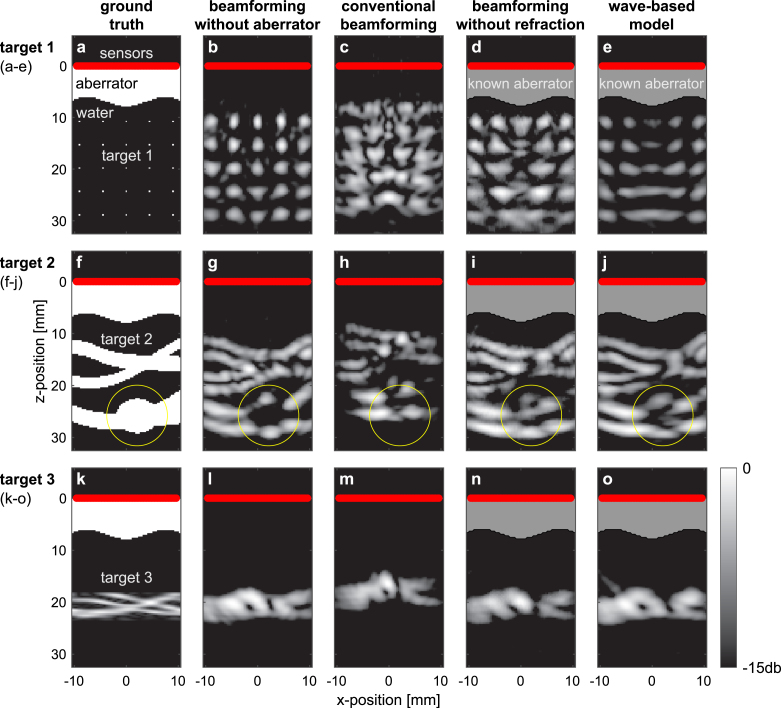
Photoacoustic imaging through a known thick aberrating layer — numerical study. **a**, Ground truth: spatial distribution of an optically absorbing target (25 white dots, target 1) behind an aberrating layer. The solid red line marks the position of a 65-detector linear array. **b**, Photoacoustic image of the target without the aberrating layer, showing the diffraction limit of the measurement system. This optimal beamforming scenario is free from distortions, offering a baseline for comparison for subsequent beamforming results, where the aberrator from panel a was introduced. **c**, Conventional (delay-and-sum) reconstruction from the aberrated ultrasound signals. The reconstruction algorithm assumes no aberrator, leading to a distorted image of the target at a wrong depth. **d**, Reconstruction when the structure of the aberrating layer is known, assuming no refraction (Eq. (4)). Incorrect image of the target’s shape appears around the true depth of the target. **e**, Our wave-based reconstruction result when the structure of the aberrating layer is known, correctly reconstructs a diffraction-limited image of the target. The curved lens-like aberrator effectively reduces the numerical aperture around x=0, thus degrading the spatial resolution achieved at that location. **f–j**, same as a–e, for a target that represents blood vessels with a rounded swelling (target 2). A yellow circle, marking the position of the rounded swelling, appears at the same position in all panels as guide to the eye. Our wave-based model (j) correctly reconstructs the rounded swelling, while showing a better overall reconstruction of the vessels when compared to beamforming without refraction (i). **k–o**, same as a–e, for a target that represents small blood vessels (target 3). The features of these vessels are smaller than the acoustic diffraction limit, leading to incorrect reconstructions of the target via all beamforming models. A better reconstruction of target 3 appears in Fig. 4.

Alternative beamforming models through thick aberrating layers typically assume a small relative difference between $c_{w}$ and $c_{a}$ [12], [13], [14], [15], thus neglecting refraction. These ‘straight-rays’ models can be expressed as:

(4) $$A^{\mathrm{ray}}\left({\vec{r}}_{s}\right)\propto \underset{n}{\sum }r_{sn}p\left({\vec{r}}_{n},t=\frac{r_{sj^{\prime }}}{c_{w}}+\frac{r_{j^{\prime }n}}{c_{a}}\right)$$

where $j^{\prime }$ is the intersection point between the aberrator’s surface and ${\vec{r}}_{sn}$, the straight line connecting the source point and the transducer element. As we show below (Figs. 2, 5), the proposed model provides improved reconstruction fidelity over both conventional DAS beamforming or the alternative beamforming of Eq. (4).

**Fig. 3:**
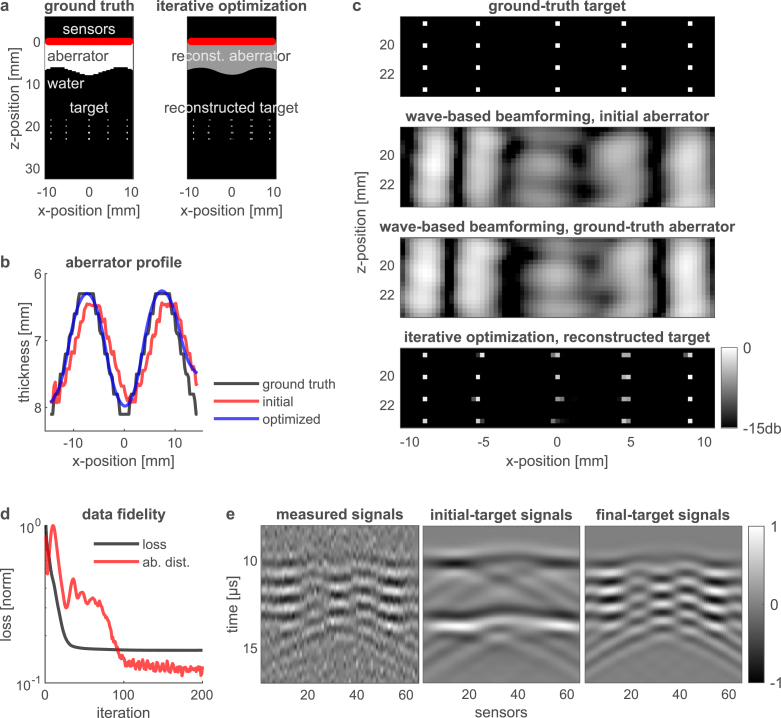
Photoacoustic imaging of sparse optically absorbing targets through an *unknown* thick aberrating layer — numerical study. **a**, Left panel: ground truth, spatial distribution of an optically absorbing target (20 white dots) behind an aberrating layer. The solid red line marks the position of a 65-detector linear array. Right panel: Our iterative optimization result, successfully retrieves both the target and the unknown structure of the aberrating layer. **b**, The optimization algorithm receives an initial, approximate guess for the aberrator’s structure as an input (red). The final, optimized aberrator (blue) is significantly closer to the ground truth aberrator (black). **c**, Zoom-in on the reconstructed target (bottom), compared to beamforming results according to our wave-based model (middle). **d**, The loss function (black) and the distance between the ground-truth aberrator and its current estimate (red), showing the convergence of the optimization algorithm. **e**, The recorded (simulated) RF signals (mean-to-mean SNR 3.0 db) compared to the signals obtained from the initial and final optimized targets based on our forward model (Eq. (1)). The final target signals accurately represent the signals simulated by the *k-wave* toolbox [25].

**Fig. 4:**
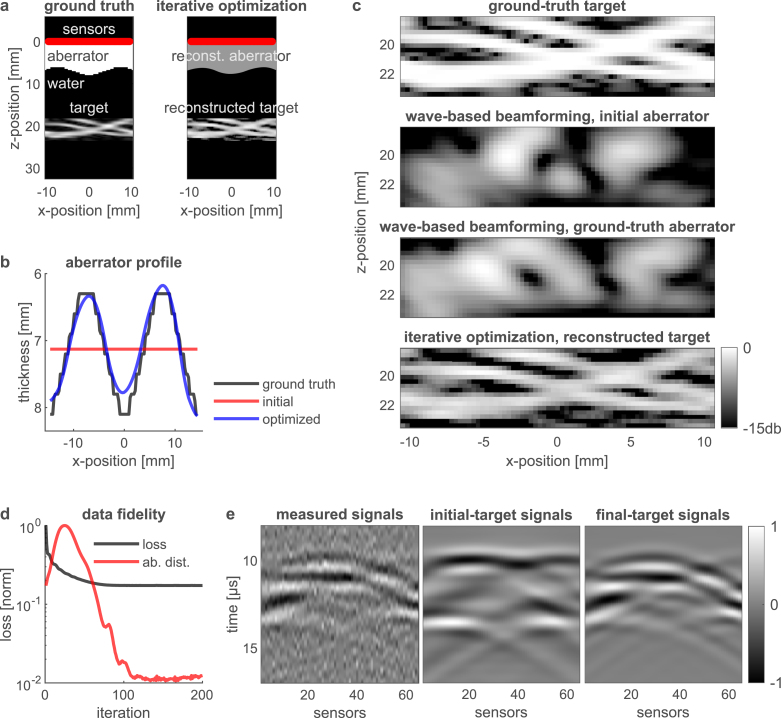
Photoacoustic imaging of a vessels-like target through an *unknown* thick aberrating layer — numerical study. **a**, Left panel: ground truth, spatial distribution of an optically absorbing target, representing small blood vessels, behind an aberrating layer. The solid red line marks the position of a 65-detector linear array. Right panel: Our iterative optimization result, successfully retrieves both the target and the unknown structure of the aberrating layer. **b**, The optimization algorithm receives an initial, approximate guess for the aberrator’s structure as an input (red). A homogeneous-thickness aberrator is assumed as an initial guess. The final, optimized aberrator (blue) is significantly closer to the ground truth aberrator (black). **c**, Zoom-in on the reconstructed target (bottom), compared to beamforming results according to our wave-based model (middle). The algorithm successfully retrieves spatial features below the acoustic diffraction limit. **d**, The loss function (black) and the distance between the ground-truth aberrator and its current estimate (red), showing the convergence of the optimization algorithm. **e**, The recorded (simulated) RF signals (mean-to-mean SNR 3.0 db) compared to the signals obtained from the initial and final optimized targets based on our forward model (Eq. (1)). The final target signals accurately represent the signals simulated by the *k-wave* toolbox [25].

### 2.3 Correcting unknown aberrators through iterative optimization

When the exact geometry of the aberrating layer is unknown, we show below that both the target and the unknown structure of the aberrator can be found through iterative joint-optimization (Figs. 3, 4, 6). The unknown parameters in this case are $\vec{\theta }=\left\{...,z_{j-1},z_{j},z_{j+1},\ldots ,a_{s-1},a_{s},a_{s+1},\ldots \right\}$, where $z_{j}$ represents the varying thickness of the aberrator above each point of the linear sensor array, and $a_{s}$ represents the amplitude of the initial pressure distribution at pixel s of the target.

The joint optimization of the aberrator profile and the initial pressure distribution is performed by an ADAM iterative gradient-descent algorithm, commonly used for the training of neural networks [26], and that has computationally efficient implementation utilizing state-of-the-art GPUs. In the ith iteration of the iterative gradient descent algorithm, the physical forward-model is calculated for the current object and aberrator parameters, ${\vec{\theta }}_{i}$, to produce the expected received signals over all transducer elements, $p^{\mathrm{model}}\left(\vec{r},t;{\vec{\theta }}_{i}\right)$. The algorithm aims to minimize the loss function, L, which consists of two terms: (1) the data consistency penalty, D, which is the difference between the expected signals $p^{\mathrm{model}}$ and the measured signals $p^{\mathrm{measured}}$, and (2) the prior penalty, P, that takes into account prior knowledge on the structure or other physical restrictions on the reconstructed aberrator parameters or absorbers distribution. The output of the gradient descent algorithm is a set of aberrator and target parameters ${\vec{\theta }}^{\mathrm{opt.}}$ that is optimized for both data consistency D and the prior P:

(5) $${\vec{\theta }}^{\mathrm{opt.}}=\mathrm{argmin}\;L\left(\vec{\theta }\right)\equiv \mathrm{argmin}\;\left\{D\left(\vec{\theta }\right)+P\left(\vec{\theta }\right)\right\}$$

where

(6) $$D\left({\vec{\theta }}_{i}\right)=\underset{n}{\sum }\underset{\tau }{\sum }{\left|p^{\mathrm{model}}\left({\vec{r}}_{n},t_{\tau };{\vec{\theta }}_{i}\right)-p^{\mathrm{measured}}\left({\vec{r}}_{n},t_{\tau }\right)\right|}^{2}$$

Here i represents the iteration number, $p^{\mathrm{measured}}\left({\vec{r}}_{n},t_{\tau }\right)$ is the acoustic signal that was measured by the sensor element n, located at ${\vec{r}}_{n}$, at time $t_{\tau }$, and $p^{\mathrm{model}}\left({\vec{r}}_{n},t_{\tau };{\vec{\theta }}_{i}\right)$ is the acoustic signal that we would expect to measure at the same location and time given the target and the aberrator parameterized by ${\vec{\theta }}_{i}$, based on our acoustic model for wave propagation (Eq. (1)).

In our experiments we defined the prior penalty P as the sum of a sparsity prior on the absorbers distribution, $P^{\mathrm{tar.}}\left(a_{s}\right)$, and the distance from the initial guess of the aberrator shape measured by ultrasound echography, $P^{\mathrm{ab.}}\left(z_{j}-z_{j}^{\mathrm{init.}}\right)$. Following the theory of compressive sampling [27], [28], we assume that the target is compressible by transform coding with a known transform, and impose sparsity of the reconstructed target shape in the corresponding transform basis by an L1-norm minimization. Specifically, for a sparse spatial distribution of absorbers, L1-norm is directly applied to the amplitude of the initial pressure distribution $a_{s}$, i.e. $P^{\mathrm{tar.}}=\left|\right|a_{s}{\left|\right|}_{1}$. Alternatively, for a target that is sparse in the Fourier domain, L1-norm is applied to the 2D Fourier transform of $a_{s}$, meaning $P^{\mathrm{tar.}}=\left|\right|F\left[a_{s}\right]{\left|\right|}_{1}$. In parallel, the mean-square-error of the aberrator profile is minimized by an L2-norm penalty: $P^{\mathrm{ab.}}=\left|\right|z_{j}-z_{j}^{\mathrm{init.}}{\left|\right|}_{2}^{2}$. All together, the prior penalty P is given by Eq. (7):

(7) $$P\left(\vec{\theta }\right)=\alpha \underset{s}{\sum }\left|{\overset{\sim }{a}}_{s}\right|+\beta \underset{j}{\sum }{\left|z_{j}-z_{j}^{\mathrm{init.}}\right|}^{2}$$

The sparsity prior penalty $P^{\mathrm{tar.}}$ is given a weight α and the penalty for changing the initial guess aberrator shape, $P^{\mathrm{ab.}}$ is given a weight β, where α and β are constant hyper-parameters that determine the magnitude of each penalty term. ${\overset{\sim }{a}}_{s}$ represents the initial pressure distribution in the appropriate transform basis.

### 2.4 Simulating photoacoustic data

To validate and test our models, we performed both numerical and experimental studies. For the numerical study, the RF-data was simulated by the *k-wave* toolbox [25], which propagated the initial pressure field (‘target’ in Fig. 2a) through heterogeneous media by iteratively solving the continuity equation (conservation of mass) and Euler’s equation (conservation of momentum), assuming a linear adiabatic equation of state. Water was simulated by the speed of sound $c_{w}=1540\;\mathrm{m/s}$ and mass density $\rho_{w}=997\;{\mathrm{kg/m}}^{3}$, and the thick aberrating layer had typical speed of sound and mass density of bone $c_{a}=2700\;\mathrm{m/s}$; $\rho_{a}=1180\;{\mathrm{kg/m}}^{3}$ [29]. The RF-data at the locations of the sensors (red line in Fig. 2a) was filtered with a Gaussian filter, centered at 1 MHz with 75% bandwidth, typical values for medical ultrasound transducers used in our experiments. A Gaussian noise with mean-to-mean signal-to-noise ratio (SNR) of 3.0 db, corresponding to noise intensity equal to 50% signal intensity, was added to all signals before applying reconstruction algorithms. The effect of different noise levels on algorithm performance is presented in Appendix B.

### 2.5 Experimental work

To experimentally test and validate our model we have performed a set of controlled laboratory experiments in a water tank. The optical source was a pulsed laser (InnoLas Inno P1864, 6 ns pulse duration, 100 Hz repetition rate, 670 nm wavelength, 25 mJ per pulse), was used for optical generation of acoustic waves. A 128-detector linear array (Verasonics L12-3V transducer) connected to a 256 channels high frequency research ultrasound system (Verasonics Vantage 256) was used for ultrasound echography and photoacoustic acquisitions. The target absorbers were realized by a set of black nylon wires with a diameter of 0.20mm (Kastking Monofilament) that crossed the imaged plane. A thick aberrating layer was realized by a shaped Perspex slab having a speed of sound of $c_{a}=2775\pm 18\;\mathrm{m/s}$, and density of $\rho_{a}=1180\;{\mathrm{kg/m}}^{3}$, similar to the average properties of bones [29].

**Fig. 5:**
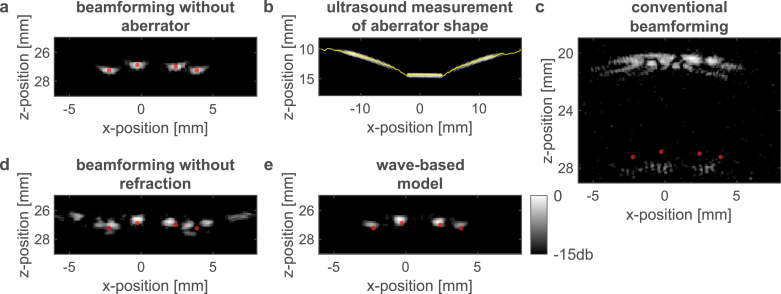
Experimental photoacoustic imaging through a thick aberrating layer. **a**, Photoacoustic image of the target without the aberrating layer, showing the diffraction limit of the measurement system. **b**, A single ultrasound transmit–receive acquisition and a conventional beamforming algorithm were used to obtain an approximate aberrator shape (yellow). **c**, Conventional (delay-and-sum) reconstruction from the aberrated photoacoustic signals, assuming no aberrator. A severely aberrated image of the target appears at the wrong depth. **d**, Reconstruction with an approximate aberrator shape (yellow in b), assuming no refraction (Eq. (4)). Incorrect image of the target’s shape appears around the true depth of the target. **e**, Our wave-based reconstruction result using an approximate aberrator shape (yellow in b), showing a near aberration-free image of the target. Red dots appear at the same positions in all panels as guide to the eye.

**Fig. 6:**
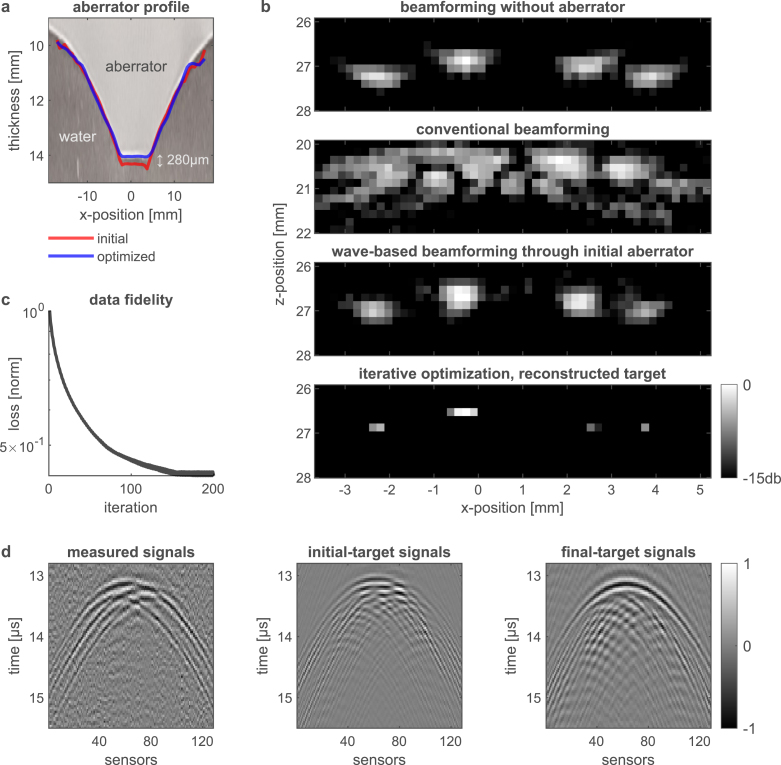
Photoacoustic imaging through an *unknown* thick aberrating layer — experimental results. **a**, An optical image of the Perspex aberrator used in this study, shown together with the initial guess (Fig. 5b) and final optimized aberrator profiles. The final, optimized aberrator (blue) is closer to the ground truth aberrator. **b**, Comparison between beamformed images of the target (top 3, same as Fig. 5a, c, e) and final reconstructed target via iterative optimization. **c**, The loss function vs. the iteration number, showing the convergence of the optimization algorithm. **d**, The recorded RF signals compared to the signals obtained from the initial and final optimized targets based on our forward model (Eq. (1)).

## 3 Results

### 3.1 Numerical results

In this numerical study we demonstrate the impact of a thick aberrating layer on photoacoustic imaging. First, we compare the effects of various assumptions about the aberrating layer and the reconstruction approach on the final reconstructed image when the exact shape of the aberrating layer is known (Fig. 2). Second, we extend the numerical study presented in Fig. 2 to scenarios where the profile of the aberrating layer is unknown, and demonstrate how both the target and the aberrator can simultaneously be retrieved via iterative optimization (Figs. 3, 4).

#### 3.1.1 Beamforming through a known thick aberrating layer

Each row in Fig. 2 represents a comparison between different beamforming approaches tested on a different target.

Fig. 2a presents the ground-truth aberrator and a target consisting of 25 small optical absorbers (target 1). The result of conventional DAS beamforming without an aberrating layer is shown in Fig. 2b. This unobstructed view illustrates the diffraction limit of the imaging system, offering a baseline against which to compare subsequent beamforming models.

Fig. 2c presents the results of conventional DAS beamforming when the aberrating layer is introduced but when the DAS beamforming assumes a homogeneous medium. This results in a severely aberrated image of the targets, with the targets appearing at an incorrect depth and shape. This image provides evidence of the challenges inherent to imaging through an aberrating layer using traditional methods.

In Fig. 2d, the known structure and speed-of-sound of the aberrating layer is taken into account in a ‘straight-ray’ beamforming reconstruction (Eq. (4)) [12], [13], [14], [15], which neglects the effects of refraction and diffraction. The image shows an incorrect shape for the targets, although they appear around the correct depth, demonstrating a modest improvement from the conventional DAS reconstruction.

Finally, in Fig. 2e, we demonstrate our wave-based reconstruction method, assuming knowledge of the aberrating layer’s structure. In this case, the reconstruction correctly reveals a diffraction-limited image of the targets. The result clearly illustrates the potential of our proposed method for accurately imaging through a known aberrating layer, offering a significant improvement over conventional techniques.

Importantly, the wave-based beamforming of target 1 in Fig. 2e maps the diffraction limit obtained by this approach. Refraction induced by the curved lens-like aberrator in Fig. 2 effectively reduces the numerical aperture around x=0, thus degrading the spatial resolution achieved at that location. This leads to a smeared image of the central absorbers (Fig. 2e) when compared to the diffraction limit without aberrator (Fig. 2b).

The second row in Fig. 2, panels f-j, extends the analysis to a different target configuration, representing blood vessels with a rounded swelling (target 2). Our wave-based model, Fig. 2j, successfully reconstructs the rounded swelling, while also showing an overall superior reconstruction of the vessels compared to the beamforming approach without considering refraction (Fig. 2i).

Lastly, in the third row of Fig. 2, panels k–o depict the same sequence for a target representing small blood vessels (target 3). These features are smaller than the acoustic diffraction limit (Fig. 2l), posing a significant challenge for all beamforming models. While all beamforming models fail to reconstruct the small features of the target, there is a clear resemblance between the reconstruction obtained by our wave-based approach, Fig. 2o, and the reconstruction obtained without an aberrator, Fig. 2l. Further improvement of the reconstruction, based on the same forward model (Eq. (1)), can be achieved via iterative regularized optimization as we show below (Figs. 3, 4).

#### 3.1.2 Photoacoustic imaging through an **unknown** thick aberrating layer via iterative optimization

In Figs. 3, 4, we present the results of our iterative joint-optimization algorithm that is capable of simultaneously reconstructing both the shape of the aberrator and the target. Fig. 3 studies photoacoustic imaging of sparse optically absorbing targets through an unknown thick aberrating layer, while Fig. 4 extends this analysis to a more complex target, representing small blood vessels imaged through an unknown thick aberrating layer.

Fig. 3a presents the ground truth: a spatial distribution of a sparse optically absorbing target (white dots) located behind a thick aberrating layer. The right panel of Fig. 3a showcases our iterative optimization result, which successfully retrieves both the target and the unknown structure of the aberrating layer. In Fig. 3b, the optimization algorithm’s process is depicted. It begins with an initial, approximate guess of the aberrator’s structure (shown in red), evolving to a final, optimized structure (blue) that closely aligns with the ground truth (black).

Fig. 3c provides a zoom-in on the reconstructed target, highlighting the differences between the beamforming results based on our wave-based model (middle two rows) and the iterative optimization result (bottom row).

The progression of the optimization is further illustrated in Fig. 3d, which plots the loss function (black) and the distance between the ground-truth aberrator and its current estimate (red). This graph demonstrates the convergence of the optimization algorithm.

Fig. 3e compares the ‘measured’ acoustic RF-signals simulated by the *k-wave* toolbox [25] with the final optimized modeled signals obtained according to our wave-based forward model, as described by Eq. (1). Notably, the final target signals closely match the signals simulated by the *k-wave* toolbox, supporting the validity of our forward model.

Fig. 4 depict the same sequence as Fig. 3, for a target representing small blood vessels. In this case, the sparsity prior (Eq. (7)) is applied to the 2D Fourier transform of the target. In addition, the optimization algorithm is starting from a homogeneous-thickness aberrator as an initial guess (red in Fig. 4b). The optimization algorithm successfully retrieves the aberrator’s structure (blue in Fig. 4b) and the smallest features of the vessels (Fig. 4c).

**Fig. B.7:**
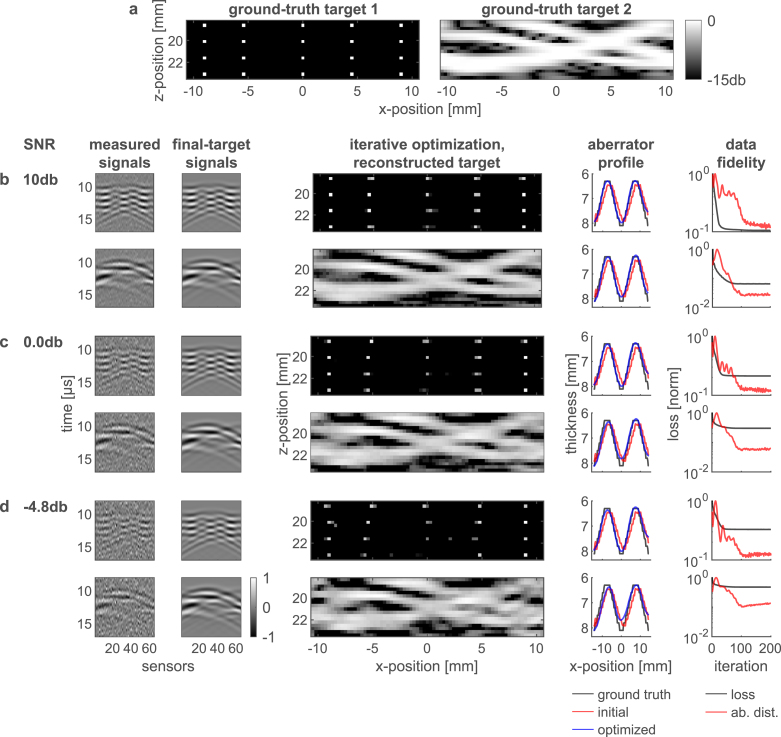
Photoacoustic imaging through an *unknown* thick aberrating layer — iterative optimization with noisy data. Each row represents a different iterative optimization run. **a**, The ground truth targets. Both target 1 (sparse points as in Fig. 3) and target 2 (vessels as in Fig. 4) are reconstructed for three different noise levels (mean-to-mean SNR 10 db, 0.0 db and −4.8 db). **b**, SNR 10 db, a reliable reconstruction of both the target and the aberrator thickness profile is obtained for both target 1 (top row) and target 2 (bottom row). **c**, SNR 0.0 db, both reconstructed targets show minor artifacts. The optimized aberrator profile starts to deviate from the ground truth aberrator for target 2. **d**, SNR −4.8 db, severely aberrated images of the targets and a less reliable reconstruction of the aberrator profiles are shown in both cases. The optimization algorithm still shows a mild improvement with respect to the initial guess aberrator profiles by reducing the distance from the ground truth aberrator in both cases.

In both Fig. 3, Fig. 4 an SNR of 3 db for the measured signals is considered. Additional optimization tests for various noise levels can be found in Fig. B.7 in Appendix B, where we study the effect of noise power on the reconstruction fidelity.

**Fig. C.8:**
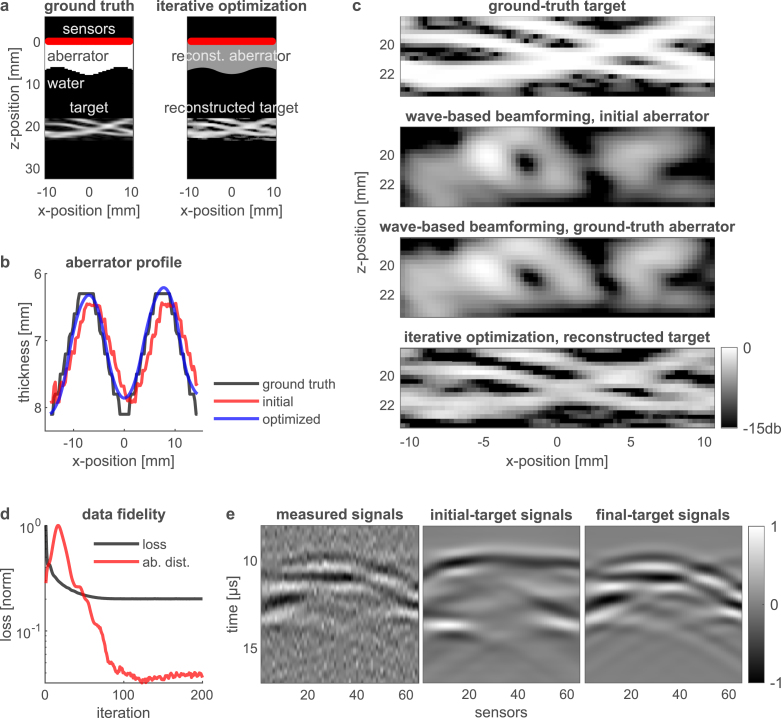
Photoacoustic imaging of a vessels-like target through an *unknown* thick aberrating layer — numerical study. **a**, Left panel: ground truth, spatial distribution of an optically absorbing target, representing small blood vessels, behind an aberrating layer. The solid red line marks the position of a 65-detector linear array. Right panel: Our iterative optimization result, successfully retrieves both the target and the unknown structure of the aberrating layer. **b**, The optimization algorithm receives an initial, approximate guess for the aberrator’s structure as an input (red). The final, optimized aberrator (blue) is significantly closer to the ground truth aberrator (black). **c**, Zoom-in on the reconstructed target (bottom), compared to beamforming results according to our wave-based model (middle). The algorithm successfully retrieves spatial features below the acoustic diffraction limit. **d**, The loss function (black) and the distance between the ground-truth aberrator and its current estimate (red), showing the convergence of the optimization algorithm. **e**, The recorded (simulated) RF signals (mean-to-mean SNR 3.0 db) compared to the signals obtained from the initial and final optimized targets based on our forward model (Eq. (1)). The final target signals accurately represent the signals simulated by the *k-wave* toolbox [25].

In Figs. 3, 4 we demonstrate that the optimization algorithm can successfully retrieve the shape of the aberrator and the target given different target and aberrator priors (sparse points vs. vessels, homogeneous vs approximate aberrator). An additional numerical investigation of the aberrator prior is presented in Appendix C, where we repeat the analysis of Fig. 4 with different aberrator initializations for various noise levels (Figs. C.8, C.9). This supplementary section demonstrates the close relation between SNR and the requirement for priors. The impact of deviations of the speed of sound estimation within the aberrator from the estimated speed of sound is detailed in Appendix D. Reverberations have a negligible effect on the reconstruction quality in the scenarios we have investigated Appendix E.

### 3.2 Experimental results

**Fig. F.12:**
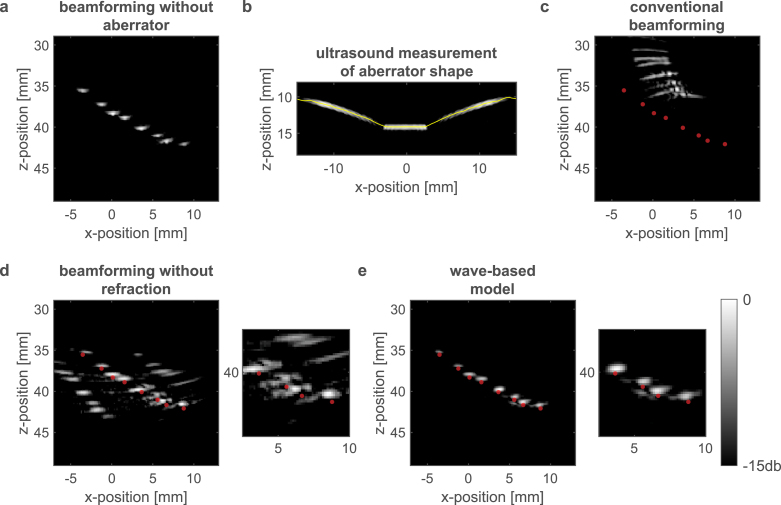
Experimental photoacoustic imaging through a thick aberrating layer. **a**, Photoacoustic image of the target without the aberrating layer, showing the diffraction limit of the measurement system. **b**, A single ultrasound transmit–receive acquisition and a conventional beamforming algorithm were used to obtain an approximate aberrator shape (yellow). **c**, Conventional (delay-and-sum) reconstruction from the aberrated photoacoustic signals, assuming no aberrator. A severely aberrated image of the target appears at the wrong depth. **d**, Reconstruction with an approximate aberrator shape (yellow in b), assuming no refraction (Eq. (4)). Incorrect image of the target’s shape appears around the true depth of the target. **e**, Our wave-based reconstruction result using an approximate aberrator shape (yellow in b), showing a near aberration-free image of the target. Red dots appear at the same positions in all panels as guide to the eye.

In the proof-of-concept experiment described below, we performed photoacoustic imaging through a thick layer of Perspex with the measured height profile given in Fig. 5b. The reconstruction results of the different beamforming approaches assuming that the aberrator shape is given by Fig. 5b are compared in Fig. 5. The results of an iterative optimization algorithm that simultaneously optimizes both the aberrator profile and the target reconstruction is presented in Fig. 6. Additional experimental beamforming results can be found in Appendix F (Fig. F.12).

#### 3.2.1 Beamforming through a **known** thick aberrating layer

A conventional photoacoustic image of the target without the aberrating layer is shown Fig. 5a. After introducing the thick aberrating layer of Perspex (Fig. 5b) between the target and the linear ultrasound array, conventional beamforming, which assumes no aberrator, results in a severely aberrated image of the target, appearing also at an incorrect depth (Fig. 5c).

To obtain an approximation of the aberrator’s shape we employ a single ultrasound transmit–receive acquisition and record the signal reflected from the distal facet of the aberrator. The speed-of-sound inside the aberrating layer, $c_{a}=2775\pm 18\;\mathrm{m/s}$, is used to obtain an image of the aberrator’s profile using a standard ultrasound delay-and-sum beamforming algorithm. A matched filter is applied to extract the approximate aberrator shape from its image (yellow line in Fig. 5b). It is worth noting that reverberations, typically present in ultrasound images of layered structures, are absent in Fig. 5b. While reverberations do appear in the raw echographic signals from which Fig. 5b was beamformed, they can be accounted for in a straightforward manner in the case of thick, homogeneous aberrating layers, as the reverberating reflections arrive to the sensor array at different times, with the first reflection also possessing a higher amplitude compared to the later reflections. Since the reverberating reflections result in beamformed deeper-lying ‘ghost’ layers, they can be naturally filtered or ignored by beamforming a small range of interest around the expected mean aberrator thickness.

In Fig. 5d, e we use the approximated aberrator shape in the reconstruction processes. ‘Straight-ray’ reconstruction without considering the effects of refraction, as described by Eq. (4), results in an incorrect image of the target’s shape, albeit located around the true depth of the target (Fig. 5d). In contrast, our wave-based reconstruction approach (Fig. 5e) shows a near aberration-free image of the target, providing an experimental validation of the robustness and accuracy of our proposed method.

#### 3.2.2 Photoacoustic imaging through an **unknown** thick aberrating layer via iterative optimization

To further refine the reconstruction of both the target and the aberrator profile, the approximate height profile of the aberrating layer as retrieved from ultrasound echography (yellow line in Fig. 5b) is used as an initial guess $z^{\mathrm{init.}}$ for our iterative optimization algorithm (red line in Fig. 6a).

In Fig. 6a, we present an optical image of the aberrator utilized in the study, in conjunction with the initial guess (red) and the final optimized shapes of the aberrator (blue). The final optimized aberrator is closer to the actual aberrator, with the main improvement appearing around the center of the linear ultrasound array (x=0). The optimized shape of the aberrator at this position is thinner by ∼280μm. For comparison, the acoustic wavelength in perspex, for the central frequency of the linear ultrasound array, is 368μm.

Fig. 6b provides a direct comparison between the beamformed images in Fig. 5 and the final reconstructed target achieved via iterative optimization. A clean reconstruction of the target is obtained even given the experimental signal-to-noise ratio (SNR) of the measured data (mean-to-mean raw SNR of the RF signals of ∼8.8 dB, Fig. 6d).

## 4 Discussion

In this study, we have presented a framework for a physics-model-based photoacoustic image reconstruction through an unknown thick aberrating layer, demonstrating its potential in both numerical studies and experimental setups. Our approach relies on a physical forward model for wave propagation that takes into account diffraction (and refraction) and can compensate for severe wave distortions induced by a single thick aberrating layer. Further, iterative optimization allows to retrieve both the unknown structure of the aberrating layer and the optically absorbing target, simultaneously.

After obtaining the optimal aberrator and target parameters, the parameters of the model can be used to correct the aberrated image to form a sharp image as would be obtained without the aberrator, or to focus ultrasound waves through the aberrator, as is used in ultrasound-based therapy [30].

Several factors are contributing to the deviations of the approximate aberrator shape from the true aberrator shape (Fig. 5b): (1) uncertainty in the speed-of-sound of the different media, (2) the acoustic diffraction limit, and (3) limitations in obtaining the aberrator’s profile by means of image processing. Despite these challenges, our experimental results showed that the distance between the approximate and true aberrators was well within the acoustic wavelength in the tested Perspex aberrator (Fig. 6a). As a result, beamforming through the approximate aberrator produced a near-perfect (diffraction-limited) image of the target (Fig. 5e), with a reliable representation of the shape of the target, and an error in depth on order of the diffraction limit. Deviations in the aberrator shape were subsequently corrected through iterative optimization (Fig. 6a).

A key advantage of our proposed method is the fast convergence of the computationally-efficient gradient-descent optimization algorithm. The optimized targets and aberrators in Figs. 3, 4, 6 were obtained within seconds in all cases. Computation times ranged from 100 to 250 ms per iteration, with the final target and aberrator appearing within 100 iterations in all cases. This raises interesting possibilities for practical applications as well as post-processing. Additional information regarding the number of target pixels, aberrator profile parametrization and numerical grid sizes can be found in Appendix G. We note that applying the proposed model and reconstruction algorithm on 3D images is possible at the price of increased computational cost and a larger memory requirement. In our current GPU implementation, the memory constraint is currently the limiting factor in reconstructing a very large number of voxels.

Our forward model can also be adapted for ultrasound imaging by incorporating an additional sum over the aberrator’s surface for the transmitted signal, expanding its potential utility.

The results presented in this paper depict the aberrator-sensors interface as a flat surface. Nonetheless, the forward model in Eq. (1) defines the pressure recorded at an arbitrary location ${\vec{r}}_{n}$, and thus it is applicable to any shape of the aberrator’s surface. Non-contact measurements can be accounted for by numerically propagating the measured signals from the measurement surface to the aberrator surface [31]. This procedure requires measuring the position and shape of the nearest aberrator surface, for example, by a single ultrasound transmit–receive acquisition followed by a conventional beamforming algorithm (similarly to Fig. 5b). This approach can be further extended to treat multiple aberrating layers of known speed-of-sound at the price of an additional computation cost.

While our iterative optimization algorithm showed promising results, it is worth noting some inherent limitations. Specifically, the nature of the optimization problem that involves multiple different physical parameters (pressure amplitude and aberrator height profile) challenges balancing convergence rates of the target and aberrator. Future work could focus on developing strategies for addressing these limitations, potentially through advanced optimization techniques or customized algorithms.

Finally, our iterative optimization algorithm lays the groundwork for physics-based learning through algorithm unrolling [32], [33]. This could further improve the performance of our proposed method, accelerating convergence speed, improving robustness, and expanding its utility across a broader range of applications.

In conclusion, this study introduces a physics-based computational framework for photoacoustic imaging through an unknown thick aberrating layer. The results of our numerical and experimental studies affirm the promise of this approach, while also highlighting avenues for further optimization and development.

## CRediT authorship contribution statement

**Yevgeny Slobodkin:** Conceptualization, Methodology, Software, Validation, Formal analysis, Investigation, Data curation, Writing – original draft, Writing – review & editing, Visualization. **Ori Katz:** Conceptualization, Methodology, Validation, Writing – review & editing, Supervision, Funding acquisition.

## Declaration of competing interest

The authors declare that they have no known competing financial interests or personal relationships that could have appeared to influence the work reported in this paper.

## Figures and Tables

**Fig. C.9:**
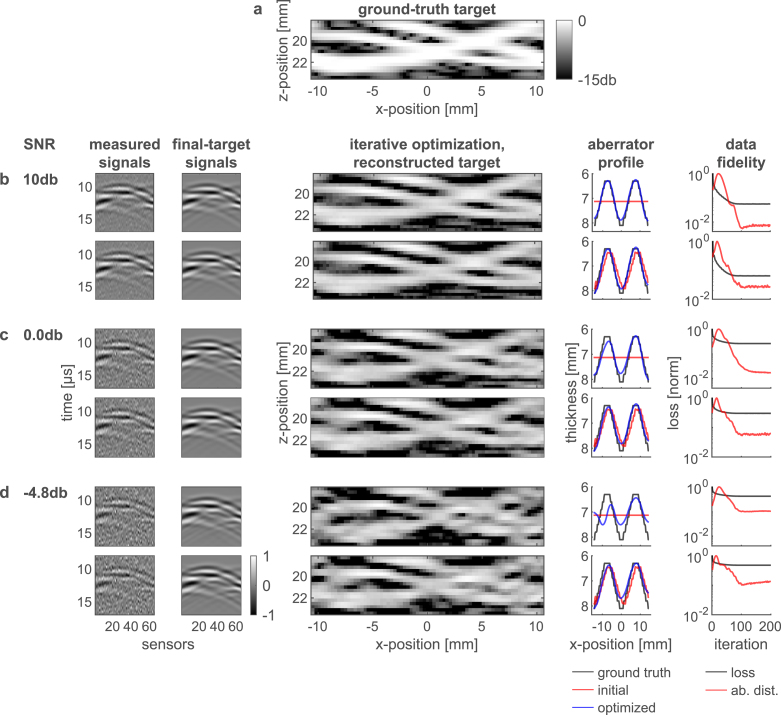
Photoacoustic imaging through an *unknown* thick aberrating layer — aberrator profile prior vs SNR. Each row represents a different initialization of the iterative optimization algorithm. Three different noise levels (mean to mean SNR 10 db, 0.0 db and −4.8 db) and two aberrator-profile initial guesses are tested. For each noise level, top row represents initialization with a homogeneous-thickness aberrator (as in Fig. 4) while the bottom row corresponds to initialization with the approximate aberrator profile (as in Fig. C.8). **a**, the ground truth target, representing small vessels (same as in Figs. 4, C.8) **b**, SNR 10 db, a reliable reconstruction of both the target and the aberrator thickness profile is obtained regardless of the initial aberrator guess. **c**, SNR 0.0 db, minor artifacts appear on the reconstructed targets. The optimized aberrator profile starts to deviate from the ground truth aberrator for when the algorithm is initialized with a homogeneous-thickness aberrator profile (top row). **d**, SNR −4.8 db, severely aberrated images of the target and a less reliable reconstruction of the aberrator profile is obtained in both cases. The reconstructed aberrator poorly represents the ground truth aberrator when the algorithm is initialized with a homogeneous aberrator profile (top row).

**Fig. D.10:**
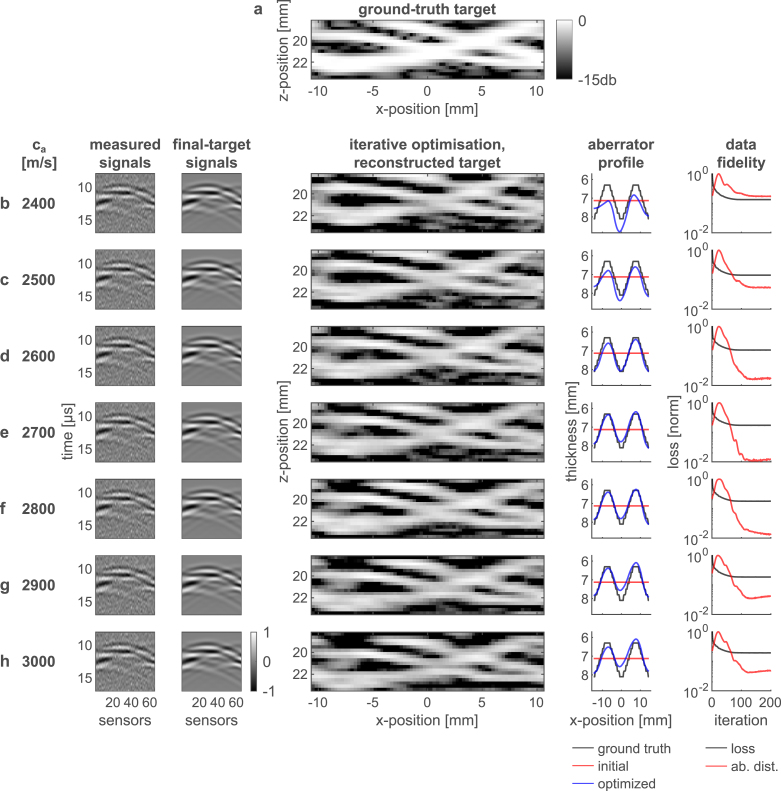
Photoacoustic imaging through an *unknown* thick aberrating layer — effect of aberrator’s speed of sound estimate ($c_{a}$) on the iterative reconstruction. Each row represents the reconstruction results when the optimization algorithm is initialized with a homogeneous-thickness aberrator and assumes a different, incorrect, speed of sound of the aberrating layer. **a**, The ground truth target, representing small vessels. **b**, Assuming $c_{a}=2400\;\mathrm{m/s}$, representing an error of ∼11% with respect to the ground truth speed of sound of the aberrator (2700m/s). To compensate for the slower speed of sound, the algorithm converges to a thicker aberrator profile and a reconstructed target that is shifted in the z-position closer to the sensor array. **c-h**, Assuming $c_{a}=2500,2600,2700,2800,2900\text{and}3000\;\mathrm{m/s}$, respectively, with panel e representing the accurate (ground truth) speed of sound of the aberrator. Errors below 10% in the speed of sound estimation predominantly influence the reconstructed target’s depth (z-position) without significantly distorting its shape. Mean-to-mean SNR 3.0 db.

**Fig. E.11:**
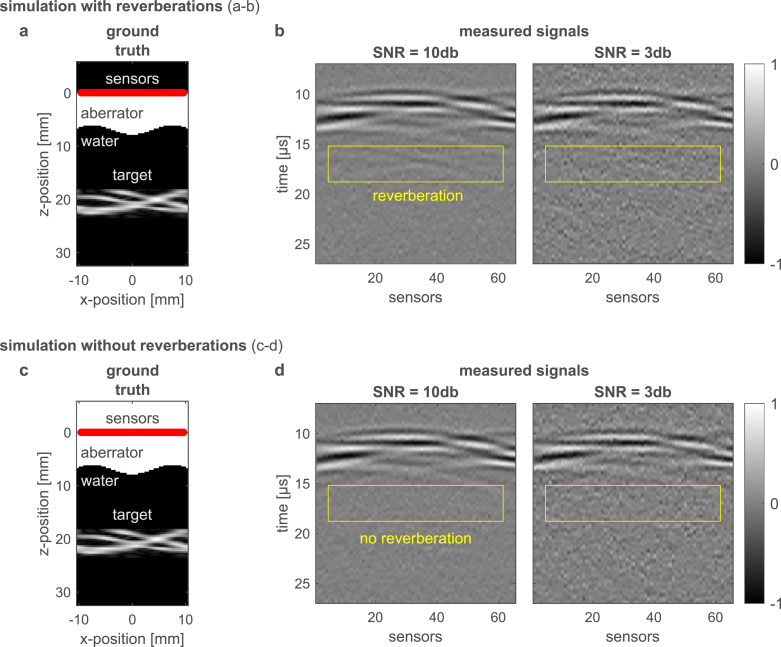
Impact of reverberations in photoacoustic RF signals. **a**, Ground truth: spatial distribution of an optically absorbing target (small vessels) behind an aberrating layer. The photoacoustic signal passes through two facets of the aberrating layer before reaching the sensors (represented by a solid red line). **b**, Recorded RF signals with varied noise levels. The presence of the first-arriving reverberation is marked by the yellow rectangle. Only the first-arriving reverberation is visible at an SNR of 10 db (left side), whereas at an SNR of 3 dB, reverberations are not visibly detectable (right side). **c**, Modified signal pathway, which mirrors the scenario in a, but with the transmitted photoacoustic signal passing through a single facet of the aberrating layer. This modification eliminates all reverberations in the recorded signal, enabling a clearer identification of the signal associated with reverberations. **d**, Corresponding to b, this part presents the signals free from reverberations. There are no discernible differences between signals with and without reverberations at an SNR of 3 db.

## Data Availability

All relevant data is available as supplementary materials in Appendix H.

## Appendix A Derivation of the forward model

Our model is based on Kirchhoff’s diffraction formula, which gives an expression for the wave disturbance when a monochromatic spherical wave passes through an opening in an opaque screen. In this chapter we follow the derivation of Kirchhoff’s diffraction formula, and adapt it to the broad-band photoacoustic signal and non-homogeneous media.

Kirchhoff’s integral theorem uses Green’s identities to derive the solution to the homogeneous wave equation at an arbitrary point $r_{n}$ in terms of the values of the solution of the wave equation and its first order derivative at all points on an arbitrary surface which encloses point $r_{n}$. The solution provided by the integral theorem for a monochromatic source is:

(A.1) $$U\left(r_{n}\right)=\frac{1}{4\pi }\int_{\sigma }\left[U\frac{\partial }{\partial N_{j}}\left(\frac{e^{ikr_{jn}}}{r_{jn}}\right)-\frac{e^{ikr_{jn}}}{r_{jn}}\frac{\partial U}{\partial N_{j}}\right]dr_{j}$$

where U is the complex amplitude of the disturbance at the surface σ which encloses point $r_{n}$, k is the wavenumber, and $r_{jn}$ is the distance from $r_{n}$ to point $r_{j}$ on the surface (Fig. 1). The derivative $\partial U/\partial N_{j}\equiv \vec{\nabla }U\cdot {\overset{ˆ}{N}}_{j}$ represents the projection of $\vec{\nabla }U$ onto the (inward) normal to the surface at point $r_{j}$ (${\overset{ˆ}{N}}_{j}$ in Fig. 1).

Assuming the media can be divided into two regions with two different (but constant) sound velocities, $c_{w}$ in water and $c_{a}$ in the aberrating layer (as illustrated in Fig. 1), we choose the surface σ to follow the interface of the two regions. In other words, we assume free space propagation in water from the source s to $r_{j}$, and free space propagation inside the aberrating layer from $r_{j}$ to $r_{n}$.

Considering a monochromatic point source at point $r_{s}$, which illuminates point $r_{j}$, the complex amplitude of the disturbance at a distance $r_{sj}$ is given by $U\left(r_{j}\right)=a_{s}\exp\left(ik_{w}r_{sj}\right)/r_{sj}$ where $a_{s}$ represents the magnitude of the disturbance at the point source, and $k_{w}=\omega /c_{w}$ is the wavenumber in water.

The projection of $\vec{\nabla }U$ onto the normal to the surface at point $r_{j}$ is therefore

(A.2) $$\frac{\partial U\left(r_{j}\right)}{\partial N_{j}}=\frac{a_{s}e^{ik_{w}r_{sj}}}{r_{sj}}\left(ik_{w}-\frac{1}{r_{sj}}\right)\cos\left({\overset{ˆ}{N}}_{j},{\vec{r}}_{sj}\right)$$

where $\cos\left({\overset{ˆ}{N}}_{j},{\vec{r}}_{sj}\right)={\overset{ˆ}{N}}_{j}\cdot {\overset{ˆ}{r}}_{sj}$. Similarly,

(A.3) $$\frac{\partial }{\partial N_{j}}\left(\frac{e^{ik_{a}r_{jn}}}{r_{jn}}\right)=\frac{e^{ik_{a}r_{jn}}}{r_{jn}}\left(ik_{a}-\frac{1}{r_{jn}}\right)\cos\left({\overset{ˆ}{N}}_{j},-{\vec{r}}_{jn}\right)$$

Here $k_{a}=\omega /c_{a}$ is the wavenumber in the aberrating layer, and $\cos\left({\overset{ˆ}{N}}_{j},-{\vec{r}}_{jn}\right)=-{\overset{ˆ}{N}}_{j}\cdot {\overset{ˆ}{r}}_{jn}$. Assuming the distances $r_{sj}$ and $r_{jn}$ are many acoustic wavelengths, i.e. $k_{w}r_{sj}\gg 1$ and $k_{a}r_{jn}\gg 1$, Eq. (A.2) is simplified to

(A.4) $$\frac{\partial U\left(r_{j}\right)}{\partial N_{j}}\approx i\frac{a_{s}k_{w}e^{ik_{w}r_{sj}}}{r_{sj}}\cos\left({\overset{ˆ}{N}}_{j},{\vec{r}}_{sj}\right)$$

Similarly, Eq. (A.3) becomes

(A.5) $$\frac{\partial }{\partial N_{j}}\left(\frac{e^{ik_{a}r_{jn}}}{r_{jn}}\right)\approx i\frac{k_{a}e^{ik_{a}r_{jn}}}{r_{jn}}\cos\left({\overset{ˆ}{N}}_{j},-{\vec{r}}_{jn}\right)$$

Plugging Eq. (A.4)–(A.5) into Eq. (A.1), we get (A.6)

$$U\left(r_{n}\right)\;=-\frac{a_{s}}{4\pi }\int_{\sigma }\frac{e^{i\left(k_{w}r_{sj}+k_{a}r_{jn}\right)}}{r_{sj}r_{jn}}$$

$$\;\;\;\;\left[k_{w}\cos\left({\overset{ˆ}{N}}_{j},{\vec{r}}_{sj}\right)+k_{a}\cos\left({\overset{ˆ}{N}}_{j},{\vec{r}}_{jn}\right)\right]dr_{j}=-\frac{a_{s}}{4\pi }\int_{\sigma }\frac{i\omega e^{i\omega \left(r_{sj}/c_{w}+r_{jn}/c_{a}\right)}}{r_{sj}r_{jn}}$$

$$\;\;\;\;\left[\frac{\cos\left({\overset{ˆ}{N}}_{j},{\vec{r}}_{sj}\right)}{c_{w}}+\frac{\cos\left({\overset{ˆ}{N}}_{j},{\vec{r}}_{jn}\right)}{c_{a}}\right]dr_{j}$$

Assuming a broadband photoacoustic signal, Fourier transform of Eq. (A.6) gives the solution in the time domain (A.7)

$$U\left(r_{n},t\right)=\frac{a_{s}}{4\pi }\int_{\sigma }\frac{\frac{d}{dt}\delta \left(t-\frac{r}{c_{w}}-\frac{s}{c_{a}}\right)}{r_{sj}r_{jn}}$$

$$\left[\frac{\cos\left({\overset{ˆ}{N}}_{j},{\vec{r}}_{sj}\right)}{c_{w}}+\frac{\cos\left({\overset{ˆ}{N}}_{j},{\vec{r}}_{jn}\right)}{c_{a}}\right]dr_{j}$$

Replacing $\frac{d}{dt}\delta \left(t-r_{sj}/c_{w}-r_{jn}/c_{a}\right)$ in Eq. (A.7) with a more general temporal impulse-response of sensor element n, $h_{n}\left(t-r_{sj}/c_{w}-r_{jn}/c_{a}\right)$, and summing over multiple point sources ($a_{s}\to \sum_{s}a_{s}$), leads to the forward model expressed in Eq. (1) of the main text.

## Appendix B Iterative optimization performance vs SNR

Fig. B.7 provides an evaluation of the performance of our iterative optimization algorithm for photoacoustic imaging through an unknown thick aberrating layer under various noise levels. The algorithm is tested for both targets of Fig. 3 and Fig. 4 of the main text (target 1 and 2 in Fig. B.7a, respectively). These targets are reconstructed across three different noise levels, with mean-to-mean SNR of 10 dB (Fig. B.7b), 0.0 dB (Fig. B.7c), and −4.8 dB (Fig. B.7d), showcasing the algorithm’s adaptability to varying noise conditions.

In Fig. B.7b, where the SNR is at 10 dB, the algorithm demonstrates its robustness with a reliable reconstruction of both the target and the aberrator thickness profile. This is evident for both target 1 (displayed in the top row) and target 2 (in the bottom row), indicating high fidelity in the presence of relatively low, yet experimentally common, noise levels.

In Fig. B.7c, with an SNR of 0.0 dB, we observe the introduction of minor artifacts in the reconstructed images of both targets. Notably, for target 2, the optimized aberrator profile begins to slightly deviate from the ground truth.

Fig. B.7d represents the most challenging scenario with an SNR of −4.8 dB. Here, the images of the targets are significantly more aberrated, and the reconstruction of the aberrator profiles is less reliable. Despite these challenges, the optimization algorithm still manages to show a mild improvement over the initial guess aberrator profiles. This is demonstrated by the reduced distance from the ground truth aberrator in both cases, highlighting the algorithm’s resilience even in highly noisy environments. In addition, the algorithm succeeds in reconstructing some key features of the targets in both cases.

## Appendix C Effect of initial aberrator profile estimate on the iterative reconstruction

In this section we test the performance of the iterative optimization algorithm for different initial estimates of the aberrator profile, used as a prior in the iterative reconstruction algorithm. Fig. C.8 repeats the analysis of Fig. 4 of the main text, when the optimization algorithm starts with an initial, close approximate guess of the aberrator’s structure (red in Fig. C.8b). The use of this close initial guess converges to a result that closely matches the result presented in Fig. 4, where the algorithm is initiated from homogeneous-thickness aberrator profile, validating the algorithmic convergence to the true result when the SNR is sufficiently high.

Next, we test the impact of initial aberrator profile on reconstruction performance under different noise levels. Fig. C.9 is structured to demonstrate how different initializations of the iterative optimization algorithm influence the reconstruction result under three noise levels (mean-to-mean SNR of 10 dB, 0.0 dB, and −4.8 dB). Two types of aberrator-profile initial guesses are tested: a homogeneous-thickness aberrator (as in Fig. 4) and an approximate aberrator profile (as in Fig. C.8).

In Fig. C.9b, under an SNR of 10 dB, the algorithm demonstrates robustness by achieving a reliable reconstruction of both the target and the aberrator thickness profile, regardless of the initial aberrator guess. This suggests that at high SNR levels, the initial guess of the aberrator profile has a limited impact on the reconstruction quality.

In Fig. C.9c, with an SNR of 0.0 dB, minor artifacts become noticeable in the reconstructed targets. Notably, when the algorithm is initialized with a homogeneous-thickness aberrator profile (top row), the optimized aberrator profile starts to significantly deviate from the ground truth. This indicates an increased sensitivity to the initial aberrator profile under moderate noise conditions.

Fig. C.9d presents the scenario with an SNR of −4.8 dB. In both cases, the images of the targets are aberrated, and the reconstruction of the aberrator profile is less reliable. Particularly, when initialized with a homogeneous aberrator profile (top row), the reconstructed aberrator poorly represents the ground truth. This highlights the critical role of the initial aberrator guess in highly noisy environments.

## Appendix D Effect of aberrator speed of sound estimate on the reconstruction fidelity

This section studies the effects of inaccurately estimating the speed of sound in the thick aberrating layer on the fidelity of reconstructed target.

Fig. D.10 presents the iterative optimization algorithm performance under varied assumptions of the aberrator’s speed of sound. The iterative reconstruction is initialized with a homogeneous-thickness aberrator profile, i.e., when both the initial shape and speed of sound of the aberrator are poorly estimated. The reconstructions reveal that, as expected, underestimating the speed of sound leads to a thicker reconstructed aberrator profile, and a target positioned closer to the sensor array. Overestimations of the speed of sound result in inverse effects. Errors below 10% in the speed of sound estimation predominantly influence the reconstructed target’s depth without significantly distorting its shape. These findings suggest that the algorithm is relatively insensitive to variations in the estimated speed of sound within the aberrator, in the tested geometries.

One can analytically estimate the expected shift in reconstructed depth for a given error in the speed of sound estimation for the case of a fixed aberrator thickness. For an estimated speed of sound $\overset{\sim }{c_{a}}$ and the correct speed of sound $c_{a}$, the resulting temporal shifts in the signal can be expressed as $\Delta t=w\left({\overset{\sim }{c_{a}}}^{-1}-c_{a}^{-1}\right)$, where w represents the thickness of the aberrating layer. These temporal signal shifts lead to an error in the reconstructed target’s depth according to $\Delta z=c_{w}\Delta t=c_{w}w\left({\overset{\sim }{c_{a}}}^{-1}-c_{a}^{-1}\right)$. Given the acoustic properties and dimensions of the tested aberrator ($c_{a}=2700\;\mathrm{m/s}$, $c_{w}=1540\;\mathrm{m/s}$, w≈1cm), Δz is expected to be smaller than the acoustic wavelength (1.54 mm in water at 1MHz) when considering up to 30% error in the assumed aberrator’s speed of sound.

While it is in principle possible to incorporate the aberrator’s speed of sound as an additional optimization parameter of the reconstruction algorithm, it comes at the price of additional hyperparameters for the optimization process, increased memory requirements, and careful balancing of convergence rates across the different variables. Given the above findings, and considering the substantial computational resources required for such an approach, the benefits of adjusting for aberrator speed of sound may not justify the added complexity.

## Appendix E Impact of reverberations in photoacoustic RF signals

RF signals passing through layered media typically undergo multiple internal reflections within each layer, known as reverberations. As in ultrasound echography, reverberating reflections in the photoacoustic signal result in reconstructed deeper-lying ‘ghost’ targets. In some cases, these ‘ghost’ targets can be filtered or ignored by reconstructing only a small range of interest around the target. In other cases, e.g., for extended targets, a ‘ghost’ image of an absorbing portion of the target may overlap a deeper target of interest. For such cases, we suggest that the signal intensity associated with reverberations is low to negligible in the imaging scenario discussed in our manuscript, as detailed below.

The power reflection coefficient for an aberrator-water interface, R, is given by

(E.1) $$R={\left|\frac{Z_{w}-Z_{a}}{Z_{w}+Z_{a}}\right|}^{2}={\left|\frac{\rho_{w}c_{w}-\rho_{a}c_{a}}{\rho_{w}c_{w}+\rho_{a}c_{a}}\right|}^{2}$$

Here, $Z_{w}$ and $Z_{a}$ denote the acoustic impedances of water and in the aberrating layer, respectively. $\rho_{w}$ and $\rho_{a}$ are the mass densities and $c_{w}$ and $c_{a}$ are the sound velocities in water and in the aberrating layer, respectively. The first-arriving reflection is expected to be attenuated by a factor of $R^{2}$ compared to the transmitted signal intensity due to undergoing two additional reflections inside the aberrating layer before reaching the sensor array. Each consecutive reverberation intensity is further attenuated by a factor $R^{2}$. Considering the acoustic properties of the aberrating layer in our paper, which resemble average properties of bones, R=0.12, and the first-arriving reverberation intensity is below 1.5% of the transmitted signal intensity. Focusing on realistic imaging scenarios with an SNR below 10 db (i.e. noise power >10% signal power), reverberations would be hardly visible in the measured photoacoustic signal (Fig. E.11). In addition, reverberations are further attenuated by absorption due to traveling a longer distance inside the aberrating layer compared to the transmitted signal.

For highly reverberating signals, a wave-based forward model can be formulated by adding an additional term that describes the specific path of each wavelet in the reverberating signal. Doing so would come at a high computational cost since it requires adding two additional sums over the two aberrator’s facets, which is analogous to introducing refraction from two additional aberrating layers.

## Appendix F Experimental beamforming

An additional experimental result, with a larger field of view and a varying distance between acoustic point-sources and the aberrator’s surface, is depicted in Fig. F.12. Similarly to the results presented in the main text, our wave-based approach (Fig. F.12e) leads to an improved reconstruction of the target when compared to conventional beamforming (Fig. F.12c) and beamforming models that neglect the effect of refraction (Fig. F.12d).

## Appendix G Algorithm implementation

In order to allow clear assessment and comparison of our proposed algorithms to prior and future work, all of the reconstruction algorithms and datasets are provided as open source, allowing to reproduce all of the presented results.

Both our wave-based beamforming and iterative optimizations algorithms can run on CPU, while the later was adapted to run on GPU. NVIDIA GeForce RTX 3090 GPU, which features 24 GB memory, was used for all iterative optimization calculations.

Key parameters regarding the implementation of the reconstruction algorithms the are listed below.

**Data preparation.** For the numerical investigation, RF-data was simulated by the *k-wave* Matlab toolbox [25], as described in Section 2.4 of the main text. Experimental data acquisition is described in Section 2.5. All beamforming and iterative optimization algorithms were applied to an IQ representation of the measured data. For simulated data, the frequency was limited to around 1 MHz, maintaining a bandwidth of 75%. In the case of experimental data, the frequency was restricted to 50% bandwidth around 7.8 MHz.

**Beamforming.** The conventional beamforming algorithm used in this paper refers to the PICMUS Matlab toolbox [34]. Both our wave-based beamforming and the straight ray (refraction-less) beamforming algorithms were coded using the same notation as in [34]. In addition to the ‘scan’ and ‘dataset’ structures, which store information regarding the target and the measured data in the original PICMUS toolbox, straight-ray beamforming and our wave-based beamforming algorithms require an additional ‘aberrator’ structure, which stores information about the aberrator’s thickness profile and speed of sound.

**Iterative optimization.** The joint optimization of the aberrator profile and the initial pressure distribution is performed by an ADAM iterative gradient-descent algorithm [26]. Our optimization algorithm is written in Python, and requires installing the following libraries: ‘torch’ and ‘numpy’ for calculations, ‘scipy’ and ‘mat73’ for saving and loading Matlab data files, ‘matplotlib’ for visualization and ‘time’ for recording computation times.

**Grid sizes and computation times.** Beamforming with simulated photoacoustic data (Fig. 2): The thickness profile of the aberrator, and the target, were sampled at 300μm intervals, using 98 points for the aberrator’s thickness profile and 15376 pixels for the target representation. The computation time for the wave-based beamforming of 15376 pixels ranged from 1401 to 1543 ms. In contrast, conventional beamforming results were achieved within 18 to 53 ms, and the beamforming results without considering refraction took between 425 to 497 ms.

Iterative optimization with simulated photoacoustic data (Figs. 3, 4, B.7, C.8, C.9, D.10): 117 points, sampled every 300μm, represented the aberrator’s thickness profile. The target was represented using 1278 pixels, with a pixel size of 300μm. The computation time for 200 iterations of the iterative optimization algorithm was 40–50 s across all simulation runs.

Beamforming with experimental photoacoustic data (Fig. 5): Both the aberrator thickness profile and the target were sampled every 100μm, utilizing 341 points for the aberrator’s thickness profile, and 5781 pixels to represent the target. Wave-based beamforming of 5781 pixels took 5427 ms, while conventional beamforming took 18 ms and beamforming without refraction took 825 ms.

Iterative optimization on experimental data (Fig. 6): 60 points sampled every 575μm represented the aberrator’s thickness profile. The experimental target was depicted using 612 pixels, with the pixel size of 175μm. Computation time for 200 iterations of the iterative optimization algorithm was 20 s.

## Appendix H Supplementary data

MMC S1 Reconstruction algorithms and datasets.
